# *Pneumocystis* Pneumonia: Pitfalls and Hindrances to Establishing a Reliable Animal Model

**DOI:** 10.3390/jof8020129

**Published:** 2022-01-27

**Authors:** Adélaïde Chesnay, Christophe Paget, Nathalie Heuzé-Vourc’h, Thomas Baranek, Guillaume Desoubeaux

**Affiliations:** 1 Service de Parasitologie-Mycologie-Médecine Tropicale, Pôle Biologie Médicale, Hôpital Bretonneau, CHRU de Tours, 2 Boulevard Tonnellé, 37044 Tours, France; guillaume.desoubeaux@univ-tours.fr; 2 Centre d’Etude des Pathologies Respiratoires (CEPR), Institut National de la Santé et de la Recherche Médicale U1100, Université de Tours, 10 Bouelvard Tonnellé, 37032 Tours, France; christophe.paget@univ-tours.fr (C.P.); nathalie.vourch@univ-tours.fr (N.H.-V.); thomas.baranek@univ-tours.fr (T.B.)

**Keywords:** *Pneumocystis* pneumonia, animal model, *Pneumocystis* spp., in vivo, infectious challenge

## Abstract

*Pneumocystis* pneumonia is a severe lung infection that occurs primarily in largely immunocompromised patients. Few treatment options exist, and the mortality rate remains substantial. To develop new strategies in the fields of diagnosis and treatment, it appears to be critical to improve the scientific knowledge about the biology of the *Pneumocystis* agent and the course of the disease. In the absence of in vitro continuous culture system, in vivo animal studies represent a crucial cornerstone for addressing *Pneumocystis* pneumonia in laboratories. Here, we provide an overview of the animal models of *Pneumocystis* pneumonia that were reported in the literature over the last 60 years. Overall, this review highlights the great heterogeneity of the variables studied: the choice of the host species and its genetics, the different immunosuppressive regimens to render an animal susceptible, the experimental challenge, and the different validation methods of the model. With this work, the investigator will have the keys to choose pivotal experimental parameters and major technical features that are assumed to likely influence the results according to the question asked. As an example, we propose an animal model to explore the immune response during *Pneumocystis* pneumonia.

## 1. Introduction

In humans, *Pneumocystis* pneumonia is a lung infection involving *Pneumocystis jirovecii*, a ubiquitous fungus with opportunistic behavior [1]. First described in malnourished children during and after World War II [2], fatal *Pneumocystis* pneumonia was one of the first signals of the Acquired ImmunoDeficiency Syndrome (AIDS) epidemic in the United States in the early 1980s [3]. The advent of antiretroviral drugs has resulted in a significant decrease in the incidence of *Pneumocystis* pneumonia in Human Immunodeficiency Virus (HIV)-positive patients. Today, in regions where HIV testing and treatment are available without restrictions, *Pneumocystis* pneumonia primarily occurs in subjects undergoing non-viral sources of immunosuppression. This includes pathological conditions responsible for the decrease in blood leucocytes such as hematological malignancies, auto-immune diseases, and drug-induced immunosuppression, such as from corticosteroids, TNF-*alpha* inhibitors, and alkylating agents [4,5]. Actually, *Pneumocystis* pneumonia occurs mainly when risk factors are cumulative (i.e., immunosuppressive therapeutic associated with a fragile medical condition). Altogether, *Pneumocystis* pneumonia affects more than 500,000 patients worldwide each year. After *Candida* spp., *P. jirovecii* is the second most common fungal agent among invasive fungal infections [6]. *Pneumocystis* pneumonia mortality is significant and has been estimated at 10–20% in HIV-positive patients and 20–40% in HIV-negative patients [7,8].

Two main forms co-exist during the *P. jirovecii* life cycle: the asci and the trophic forms, which are differentially involved. Transmission, which is human-to-human airborne, is ensured by the asci form, the only form capable of living transiently in the external environment [9,10]. Next, the trophic forms thrive at the surface of type I pneumocytes in the pulmonary alveoli. This leads to the generation of local inflammation, while the infection remains extracellular and never becomes invasive in the tissues [11,12]. The specific biological diagnosis relies on the microbiological identification of *P. jirovecii* in pulmonary secretions and lung tissues by microscopic examination and qPCR. Likewise, it can be indirectly suggested by measuring (1,3)-β-D-glucan, a polysaccharide component of the cell wall of *P. jirovecii* and other fungi, in the serum of patients [13,14].

Despite some advancements in the scientific knowledge, *Pneumocystis* pneumonia still contains many unknowns. The cycle of *Pneumocystis* is not fully elucidated yet, thus preventing from dispensing clear prevention guidelines. Concerning the pathophysiology, there is a critical need to investigate all the immune mechanisms integrated in the host response. Therefore, experimental models are essential to completing clinical studies. Although theoretically easier and sparing animal lives, in vitro models are unable to mimic the complexity of host–fungus interactions. Importantly, there is no in vitro continuous culture system for *Pneumocystis* spp., despite long research in this area [15,16]. Animal models can circumvent these limitations [17,18,19]. Therefore, various animal models of *Pneumocystis* pneumonia have been developed in attempt to address pathogenesis, virulence, immune response, diagnosis, or therapy concerns [19,20,21,22,23,24]. However, a single model cannot answer all the aforementioned questions, which explains in part the great multiplicity of supports that have been developed so far. This variability can hinder the scientific comparisons, and each mammal species has its own *Pneumocystis* species (e.g., *P. murina* for the mouse or *P. carinii* for the rat). Of all the animal model variables, the investigator has to question the pivotal experimental parameters and major technical features that are assumed to be likely to influence the results according to the question asked.

**Figure 1:**
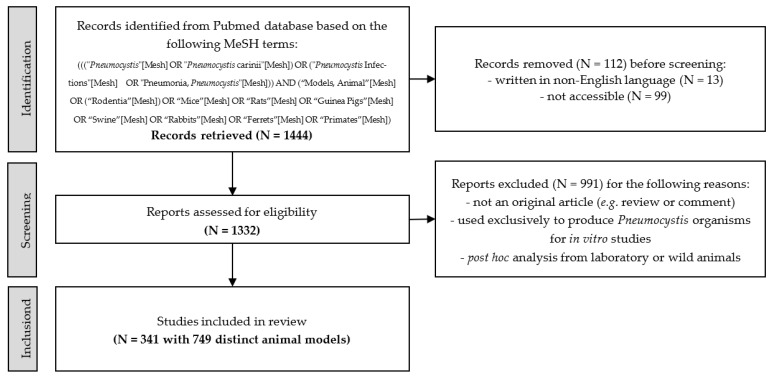
Flow chart of the bibliometric study. The research was completed in PubMed up to December 2020. Scientific reports, oral communications, and posters were not addressed in this study. N = number.

**Figure 2:**
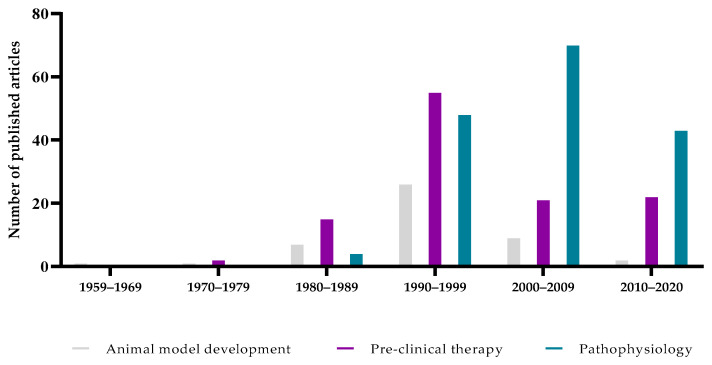
Distribution of articles according to the decades of publication and the topics. For this bar chart, only the articles about animal models of *Pneumocystis* pneumonia retrieved in PubMed up to December 2020 were considered, according to the criteria reported in Figure 1.

Here, we conducted an extensive literature review of published reports related to animal models of *Pneumocystis* pneumonia using a search strategy in the PubMed database for articles published up to December 2020, based on MeSH terms. Our electronic request about animal models of *Pneumocystis* pneumonia retrieved 1444 publications. Experimental animal studies were included when they met the following inclusion criteria: (1) the article was accessible and written in English; (2) the study was an original article; (3) the animal model was not exclusively used to produce *Pneumocystis* organisms for an in vitro study; and (4) the study was not a post hoc analysis with laboratory or wild animals. After thorough reviewing, a total of 341 articles, corresponding to 749 distinct animal models, were finally retained for complete analysis (Figure 1). Initially, the articles were mostly dedicated to the description of the implementation of animal models and to preclinical therapeutic studies. Then, at the beginning of the 2000s, pathophysiology studies became by far the largest area of experimentation (Figure 2). Initially, the development of the first animal models required dedicated articles for sharing with other experts. At the same time, these first animal models were used to test various therapeutic molecules, alone or in combination, which were already available on the market for the management of other infections. Thereafter, the therapeutic arsenal expanded a little, and the need to deepen the knowledge on the pathophysiology of *Pneumocystis* pneumonia became essential, explaining, in part, the distribution of the article topics according to time. We now propose to the reader a progressive and in-depth review of the elements that we consider essential in the design of an animal model of *Pneumocystis* pneumonia (i.e., the host species, the parameters inducing susceptibility to *Pneumocystis* pneumonia, the implementation of the experimental infection (i.e., route of inoculation and fungal inoculum) and the biological parameters to follow up to assert correct implementation of the disease).

## 2. General Description of the Various Animal Models: Host Species and Strains, Sex, Weight, and Age

The choice of the host species is critical to reproduce the pathology that develops in humans as faithfully as possible and also to ensure the best reproducibility. Indeed, as in humans, animals need to be carriers of *Pneumocystis* and transmit it to their congeners by air. Additionally, as in humans, depending on their immune status, they must be able to eliminate the fungus naturally without developing a disease if they are immunocompetent or, on the contrary, in case of immunosuppression. Overall, more than 10 animal species have been used as host models for the in vivo study of *Pneumocystis* pneumonia so far.

Unsurprisingly, rodents were extensively exploited (95.9%) compared with other orders of mammals (Table 1). Mice were used in 74.8% of the selected studies, compared with 20.8% and 0.3% for rats and other rodents (e.g., Guinea pigs and hamsters), respectively. The mouse model was widely used for its well-characterized physiology, as well as its biochemical and genetic homologies with humans [25], but also for the dedicated toolbox that has been developed. Rabbits were used in 1.3% of the studies. Nonetheless, rabbits usually display lower fungal loads than other animals, and few tools and products are adapted to the rabbit’s biology. In addition, they are more expensive and difficult to handle than rats and mice. In 1.3% of the models, non-human primates (NHPs) were used from two species belonging to the family of Cercopithecidae [26,27,28,29,30,31,32,33]. The latter, thanks to their physiological similarities and evolutionary conservation with humans, represented privileged models for studying *Pneumocystis* pneumonia in a viral immunodeficiency background. Nevertheless, even if humans and NHPs are closely related, it should be kept in mind that each is contaminated by its own species: *P. jirovecii* for humans and *Pneumocystis carinii* f. sp. *Macacae* for macaques. Other mammals were rarely used, such as ferrets [34,35,36], pigs [37,38,39], cats [37], and dogs [37]. Lastly, two arthropod-based studies, *Drosophila melanogaster* and *Galleria mellonella*, assessed the non-susceptibility of non-mammalian species to *Pneumocystis* pneumonia [40,41]. The relative benefits and limitations of the four major animal models (mouse, rat, rabbit, and NHP) for the study of *Pneumocystis* pneumonia are summarized in the Table 2.

**Figure 3:**
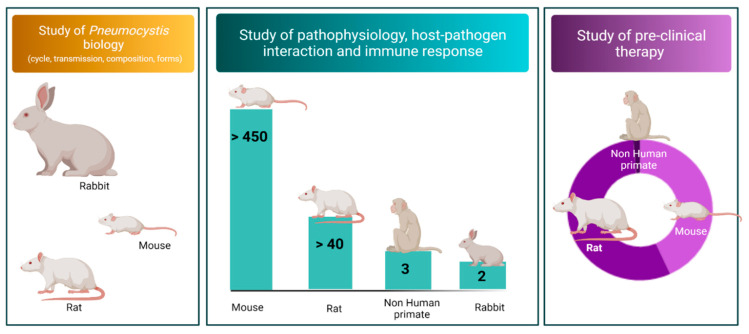
Current distribution of the main animal models and their utility in the *Pneumocystis* pneumonia study. The figure includes the four most commonly used animals in models to study *Pneumocystis* pneumonia or the *Pneumocystis* agent. The number superimposed on the bars represents a count of the studies included in our review, using a given species (PubMed search, 31 December 2020). The colorized area represents the proportion of the studies dealing with pre-clinical therapy included in our review using a given species (PubMed search, 31 December 2020).

Depending on the purpose or issue of the study, some animals were used more frequently than others (Figure 3). Rabbits have most commonly been used to study the *Pneumocystis* agent and its transmission. Indeed, spontaneous *Pneumocystis* pneumonia is described in the absence of induced immunosuppression at the time of weaning, thus naturally facilitating its study [42,43]. Mice and rats have also been used to study the transmission of *Pneumocystis* between the same or different host species. Mice have been mostly used to study host–pathogen interactions and the host’s immune response. Non-human primates have been used little, in part due to ethical restrictions. Finally, rats have been the preferred species for pre-clinical therapy studies (prophylactic, immunization, and curative).

The importance of an informed choice for the animals concerns not only the species, but also the strain (Table 1). Focusing on mouse models, studies using inbred strains predominated. BALB/c and C57BL/6 were reported more before C3H/HeN. Attention should be paid to the selection of strains, as highlighted in a study conducted by Swain et al. in which BALB/c and C57BL/6 mice were shown to develop a different specific early immune reaction after inoculation with *P. murina* [44]. The strains also appeared to show a different permissiveness to *Pneumocystis* infection with variable lung burdens, as shown by Tisdale et al. [45]. Considering all animal models other than mice, outbred animals were used more frequently than inbred ones. For studies with outbred rats, Sprague-Dawley represented 64.1% of the rat models, while the Wistar strain was associated with 14.7% of the reports. The data on susceptibility in different rat strains do not seem to be unanimous. Whereas Boylan et al. evaluated that Sprague-Dawley, Fisher 344, and Lewis rats immunosuppressed by steroids developed the same heavy infection 6 weeks after inoculation, Hong et al. showed that Wistar rats developed an earlier and more severe infection than Fisher and Sprague-Dawley rats under steroid immunosuppression [37,46].

The sex of the animal chosen is also important, although in the majority of the models (64.6%), it was not specified (Table 1). When reported, they were females in 48.3% of cases, males in 37.4% of cases, and both genders in 14.3% of cases. In a study comparing the progression of *Pneumocystis* pneumonia in males and females, Tisdale et al. showed that females of three distinct mouse strains had higher fungal burdens compared with males after 6 weeks of infection [45]. This contrasts with what is usually observed in humans, where men are the most affected by *Pneumocystis* pneumonia [47,48]. Concerning the weight of the animals used, when informed (14.2%), it was quite homogeneous and standard, being 21.0 ± 4.5 g and 189.4 ± 48.4 g for mice and rats, respectively. In models of *Pneumocystis* pneumonia, weight loss is rarely reported and appears to be a poor and irrelevant indicator of disease. Moreover, in human medicine, there are very few data on the importance of the initial weights of patients suffering from *Pneumocystis* pneumonia, with only a few cases reported in a context of nutritional deprivation [49,50]. In contrast, the choice of life stage of the animals may be an important element, especially considering that the immune system is not fully developed during the first weeks of life and strongly evolves throughout aging [51]. Indeed, studies have compared the different life stages of mice in relation to the immune response. Neonates showed a delay in the onset of the immune response due to an inadequate lung environment coupled with an inherent inability to develop a robust innate immune response to infection and an inexperienced adaptive immune system [52,53,54]. However, to our knowledge, there are no data on older animals where the immune system is undergoing age-related senescence.

## 3. Selection of the Regimen Inducing Susceptibility to *Pneumocystis* Pneumonia

In the great majority of cases, tools to render animals susceptible to *Pneumocystis* pneumonia are an essential element to consider. Indeed, patients susceptible to *Pneumocystis* infection have the particularity of presenting pre-existing underlying conditions. Therefore, usage of a regimen inducing susceptibility to *Pneumocystis* pneumonia was reported in 663 animal models (i.e., 88.5% of those described). In Table 3, we propose summarizing the advantages and disadvantages of the principal strategies to render animals susceptible to *Pneumocystis* pneumonia.

Based on analogy with other models of fungal infections of the respiratory tract (e.g., aspergillosis), anti-cancerous drugs like alkylating substances, and more specifically cyclophosphamide, were used to induce adequate immunocompromised conditions [55,56]. However, alkylating agents primarily target neutrophils, which are less involved in the response to *Pneumocystis* than T-lymphocytes and macrophages. The latter are rather targeted by steroids, recognized as a major risk factor for the development of *Pneumocystis* pneumonia [57,58,59]. They have been largely used to induce immunosuppression in animal models of *Pneumocystis* pneumonia (30.8% of the animal models) [60,61,62,63,64,65,66]. Dexamethasone administered in drinking water at a concentration of 1–4 mg/L was most commonly used (57.8% of steroid models), ahead of injectable cortisone acetate (23.9% of steroid models) and injectable methylprednisolone (15.2% of steroid models), both administered subcutaneously. Dexamethasone has the advantage of a longer duration of action but also a higher anti-inflammatory potency than cortisone and methylprednisolone. Oral administration is convenient, relatively safe, economical, and compatible with refinement of experimental procedures, although it does not possess the highest bioavailability compared with parenteral routes of administration [67]. In most models, steroid-dependent immunosuppression started 1–2 weeks prior to the experimental challenge in order to reproduce a suitable condition for the development of *Pneumocystis* pneumonia [68], and this was continuously pursued until the infection had been established [69,70]. Other immunosuppressive drugs were alternatively used in rare models: dichloromethylene diphosphonate-containing liposomes or clodronate-liposomes for the specific depletion of macrophages [66,71,72] or more broad-spectrum medicines such as calcineurin inhibitors, tacrolimus and ciclosporin [73], mTOR inhibitor, sirolimus [74], or mycophenolate mofetil, an inhibitor of inosine-5’-monophosphate dehydrogenase [74].

Considering that CD4^+^ T-lymphocytes count as a reliable predictor of opportunistic *Pneumocystis* pneumonia during HIV infection [68], a more specific treatment of this lineage has also been tested. Depleting monoclonal antibodies (mAbs) targeting CD4^+^ T-lymphocytes (clone GK1.5) were widely used (81.7% of the models based on immunotherapy) alone or in combination with other T-cell-depleting mAbs such as anti-CD8 (clone 2.43) or anti-Thy1.2 (clone 30H12) mAbs in mice. Some other antibodies were given, such as anti-CD20 mAb (clone 5D2 or 18B12), allowing B cell depletion [75,76]. mAbs could be administered either once before or just after the experimental infection or several times throughout the course of infection. Immunotherapy was most often administered by intraperitoneal injection and almost exclusively in mice. Unfortunately, the risk of hypersensitivity reaction or cytokine release-associated acute reactions and the multiplication of parenteral injections constitute major drawbacks [77,78].

Genetically modified mice also offer interesting advantages for the development of *Pneumocystis* pneumonia and have been widely used (56.4% of the studies in mouse models). They can be grossly divided into two groups: (1) models displaying a general immunodeficiency, such as Severe Combined Immunodeficiency Disease (SCID) or Recombination activating gene RAG1-/- mice that lack functional T-cells or B-cells, or (2) more refined models that target a specific gene implicated in the host response. The first ones mentioned were primarily used to study *Pneumocystis* biology, including its life cycle and the efficiency of anti-*Pneumocystis* curative drugs. In 1993, for example, the study by Chen et al. used CB17/scid (SCID) mice to support the concept that *Pneumocystis* pneumonia develops in immunocompromised patients because of recent exposure to an exogenous source and not necessarily because of reactivation of latent infection [79]. The second ones were exploited to study and identify cellular and molecular entities involved in the innate and adaptive anti-*Pneumocystis* immune responses. For example, the involvement of the surfactant proteins A and D in fighting against *Pneumocystis* was highlighted by the generation of deficient mice that were knocked out for the relative encoding genes [80,81,82,83,84,85,86]. Later in 2018, Elsegeiny et al. used several mouse models to recapitulate human primary immune disorders, enabling them to understand which types of CD4 T-cells were involved or relevant to mediating the clearance of *Pneumocystis* [19]. However, care should be taken when interpreting the outcomes in these models because of redundancy in the immune system or compensatory hyperactivity that can lead to confounding effects [87]. In addition, scientists have to keep in mind that the use of such genetically modified or defined mice under standardized environmental conditions may influence host immunity and inflammation [88]. While the generation of such mice still remains complicated, expensive, and time consuming, they represent very useful biological tools for studying the host immune response to *Pneumocystis*.

Alternative immunosuppression procedures have also been implemented. This was the case for the majority of *Pneumocystis* pneumonia models in NHPs. In order to reproduce as closely as possible the immunosuppression that affects AIDS patients, NHPs were infected intravenously with Simian Immunodeficiency Virus (SIV) [26,27,28,29,30,31,32].

To enhance the magnitude of *Pneumocystis* infection, a low-protein diet was used in 7.9% of the models [89]. This particular diet, which is harmful to longevity and metabolic health, was set up to reproduce the malnutrition status observed in some patients suffering from *Pneumocystis* pneumonia. However, it was quite expensive and barely used after the 2000s.

Since the models are mostly immunocompromised, it is important to use an antibiotic prophylactic strategy to prevent from the occurrence of opportunistic bacterial infection, which would occur more quickly than the *Pneumocystis* pneumonia. Antibiotics were used in 23.0% of the models. The molecules used belonged to a broad spectrum of antibiotic families. Cyclins were the most widely used, being in 70.9% of the models using antibiotics. Tetracycline was administered in drinking water at a concentration between 0.5 and 1 mg/mL, and doxycycline, which was by far less used, was administrated by subcutaneous injection. Beta-lactamins were used in 26.2% of the models, along with ampicillin, cephadrin, penicillin G, and amoxicillin with or without clavulanic acid. They were mostly administered in drinking water. Other antibiotics were less used, such as quinolones with ciprofloxacin [71], aminosides with streptomycin and gentamicin [90,91], or sulfamides with sulfadiazine [92]. Anecdotally, 2.4% of the models used polyenes, nystatin, or amphotericin B to prevent other fungal diseases. The antibiotic prophylaxis strategy based on the use of cyclins, especially tetracycline, which is widely used, inexpensive, and easily administered in drinking water, is to be preferred. Concerning the use of an antibiotic prophylactic strategy, the parallel with what can be observed in human medicine is complicated to establish. Indeed, while most cases of *Pneumocystis* pneumonia occur in immunocompromised patients, little or no retrospective data are available on the use of antibiotics concomitant with the development or diagnosis of *Pneumocystis* pneumonia. Such information could be of interest in assessing the impact such a treatment might have on the pathophysiology of the disease.

## 4. Implementation of the Experimental Infection

Setting up a relevant animal model of fungal infection requires considering the route of infection. Three main methods of experimental challenge have been proposed in the literature for generating *Pneumocystis* pneumonia.

A first passive strategy was based on the presumed latency of *Pneumocystis* within the lung alveoli and its subsequent reactivation following the induction of immunosuppression. This strategy was adopted in 20.7% of the models, especially in the pioneer reports. With respect to the recent evidence in favor of de novo infection, this protocol seemed clearly inadequate and, moreover, insufficient to ensure a methodologically strict and reproducible study. Indeed, in most of these ancient reports, animals were kept under unspecified exposure conditions, and the occurrence of *Pneumocystis* pneumonia was quite random and most likely due to the transmission of *Pneumocystis* organisms by the other animals housed in the same facilities. Nowadays, one acknowledges that it is essential to use animals with Specific and Opportunistic Pathogen Free (SOPF) certification in housing conditions such as microisolator-filtered cages that eliminate the risk of transmission from other animals.

A second passive strategy, used in 17.0% of the studied models, was implemented by co-housing healthy animals with *Pneumocystis*-pre-infected seeder mate fellows. Indeed, the airborne route was clearly established in the early 1980s in germ-free immunocompromised rats that had been exposed to potential sources of *Pneumocystis carinii* (i.e., natural *Pneumocystis* species in rats) [93]. In isolators, rats exposed to filtered sterile air and unsterile water and food did not acquire *P. carinii*, while animals exposed in open cages to room air but maintained on sterile diets acquired the infection. Thus, thanks to this model, it has been demonstrated that *Pneumocystis* was naturally acquired by horizontal transmission as an airborne organism in a de novo infection [69,93,94]. In the same vein, healthy immunocompromised animals were co-housed with fellows of the same species infected with *Pneumocystis* for a time varying from 1 day to several weeks [95,96,97,98,99,100]. It appeared that the inoculum or dose effect determined the rate of infection progression [101]. Although this kind of strategy replicates the natural transmission of *Pneumocystis* in mammals, it could lack control and reproducibility.

In order to control these points, a third experimental infection strategy was developed through the direct inoculation of *Pneumocystis* organisms into the animals’ respiratory tracts. Various modes of administration have been developed. Most of the time, animals were sedated or anesthetized prior to delivery in order to minimize struggling and sneezing. The anesthesia procedure and the operator skills were critical to achieve a robust and reliable infection [102]. Inoculation of *Pneumocystis* organisms could be achieved by intranasal, oropharyngeal, or intratracheal instillation or by transtracheal deposition. The intranasal instillation, consisting of the deposition of droplets of a *Pneumocystis* suspension close to the nostrils, appears to be the softest method (easiest and the least invasive technique). At the opposite end, the transtracheal alternative requires exposing the trachea surgically to a direct injection of organisms. Intratracheal delivery of *Pneumocystis* via a blunted needle or feeding cannula allows for refining of the procedure by getting rid of the surgical incision. Overall, the direct inoculation strategy was the most common method used in mouse models, with the majority of administration based on intratracheal instillation (Table 1). The frequency of *Pneumocystis* inoculation was generally based on a single administration, except for some specific studies that completed two or three successive inoculations separated by 2–20 days [52,103,104,105,106]. Garvy et al. performed several inoculations to induce immunization [52], whereas Vuk et al. used a second inoculation to be certain that the mice strains used, known to exhibit low levels of *Pneumocystis* infection according to them, were sufficiently exposed to *P. murina* organisms [106]. None of the studies compared multiple inoculations vs. a single one. Thus, it is difficult to appreciate whether this resulted in greater infection. However, the time until the onset of *Pneumocystis* pneumonia was similar, whatever the number of inoculations used. The advantages and disadvantages of each strategy to implement *Pneumocystis* pneumonia are summarized in Table 3.

Other concerns arose from the variability of the composition and the size of the *Pneumocystis* inoculum. Because, so far, in vitro production of *Pneumocystis* has not been successful, *Pneumocystis* were extracted and mostly purified from fresh or frozen pulmonary grindings of previously infected animals. Extraction could be based on different methods, such as stomacher blending, ultrasonication, or magnetic stirring [10,107,108,109]. Because *Pneumocystis* organisms can only be partially purified, the inoculum will contain immune cells, cytokines, or other immune stimulators that may affect the host’s pulmonary immune response. Thus, a control with lungs from healthy animals having undergone the same purification process seems to be essential. In some rare publications, the animal received *Pneumocystis* asci from another animal species [40,41,110,111]. Although Walzer et al. initially showed that the sporadic transmission of *Pneumocystis* was possible between rats and mice [110], the opposite was subsequently demonstrated and definitively admitted [111]. Furthermore, there was great diversity in the ways to count the number of *Pneumocystis* organisms in order to prepare the infectious suspension for the experimental challenge. When some counted only the asci through microscopic observation, others counted the trophic forms as well [82,112,113,114]. It is noteworthy that counting the trophic forms is a tedious task and requires a great deal of experience on the part of the microscopist, and taking trophic forms into account is also quite sensitive, since they were shown as insufficient to induce *Pneumocystis* pneumonia [9,10,113,115]. In a concern of homogeneity and scientific relevance, it seems more appropriate to consider and count only the asci for the inoculum. Large variations in the inoculum size, defined by the prior numbering of *Pneumocystis* forms, were observed from the 1.0 × 10^4^ to 1.0 × 10^8^ *Pneumocystis* forms, with an average from around 1.0 × 10^6^ to 1.0 × 10^7^ *Pneumocystis* organisms. Thereafter, the experimentalist should be aware that the establishment of clinical *Pneumocystis* pneumonia is a long process requiring 4–7 weeks after inoculation.

## 5. Validation of the Model and Outcome Parameters to Follow Up

In all the infectious animal models, it is essential to verify the effective infection or colonization and quantify the microorganism load. Since the clinical and radiological signatures of *Pneumocystis* are not specific, the use of histological biological techniques was almost systematic, although none of these methods provided actual information about the viability of the fungal elements. Overall, 98.4% of the articles reported at least one histological or biological test (including microscopic approaches) to confirm that the experimental infection was correctly implemented in the exposed animals or to assess the fungal burden. However, most of the models exploited only one technique (78.6%).

Microscopic observations of pulmonary secretions, lung sections, and lung grindings slides, long considered as the reference standard to prove *Pneumocystis* pneumonia or colonization, have been largely described in 81.8% of all models. These direct methods used different types of staining like Diff quick, Giemsa, Grocott methanamine silver nitrate (GMS), and toluidine blue O or calcofluor-blue brightener to demonstrate the presence of discoid *Pneumocystis* asci, ascospores, or trophic forms. Microscopic approaches require substantial microscopic expertise, but they seem essential because they allow one to distinguish the asci forms quickly while being easy-to-implement and inexpensive methods.

Methods based on molecular biology like nucleic acid amplification by qPCR or fluorescence in situ hybridization (FISH) are more sensitive techniques. They are more refined to determine the fungal load (asci and trophic forms included) and can be used in various kinds of samples (e.g., lung tissues, bronchial-alveolar lavage fluids (BALF), or oral swab samples). They were widely used in 31.8% of the models with the following targets: the mitochondrial large subunit (mtLSU) rRNA gene, mitochondrial small subunit (mtSSU) rRNA gene, 5.8 S rRNA gene, dihydrofolate reductase (DHFR) gene, and kexin-like serine protease (Kex1) gene. As for other molecular biology methods, qPCR requires specialized, costly equipment and reagents, which are now available in a large number of laboratories. It should be noted that the primers used for *Pneumocystis jirovecii* usually do not overlap with those of other *Pneumocystis* spp., like *P. murina*.

Other tools were seldom used, such as the detection of anti-*Pneumocystis* antibodies, which was performed in 6.9% of the models, or the bloodBALF detection of (1,3)-β-D-glucan [14,114,116,117,118]. The serology, which was never used alone, was attended by huge difficulties involving potential false-negative test results, and it is questionable with regard to the production of antibodies in immunocompromised animals. In humans, its use is restricted to epidemiological questions [119]. In some studies, in particular with NHPs, the authors used combinations of tools, including modification of the antibody titer associated with qPCR in BALF to discriminate infection from colonization [28,29,30,31]. The detection of (1,3)-β-D-glucan is not specific to *Pneumocystis* pneumonia and is quite costly.

In general, and whatever the type of study, to assess the presence of *Pneumocystis*, identify its forms, and ensure the most accurate quantification possible, the combination of a microscopic and molecular biology technique appears the most suitable.

## 6. Conclusions

*Pneumocystis* pneumonia is a severe respiratory disease that occurs especially in immunocompromised patients. Worldwide, the number of deaths due to *Pneumocystis* spp. is estimated to be almost 250,000 (Gaffi data, 2017). In the absence of models of continuous in vitro culture, in vivo animal studies represent a crucial cornerstone for the study of *Pneumocystis* pneumonia. However, it is important to keep in mind that *Pneumocystis* species are host-specific [35]; they progressively diverged several tens of millions of years ago and co-evolved with their hosts, thus defining their host obligate nature [120,121]. Therefore, these models are imperfect, and we can wonder about the extrapolation of the results obtained with models using microorganisms genetically different from those infecting humans.

Ethical considerations are important when planning the use of an animal model and should be governed by the “3 Rs” rule: replacement, reduction, and refinement [122]. Animal experiments should be designed in such a way that they allow statistically significant results with the smallest possible number of animals while being robust and reproducible. In such a manner, the choice of the animal species and strains for studying *Pneumocystis* pneumonia is decisive. As seen previously, the mouse seems to be the most suitable species. Refinement in animal models of *Pneumocystis* pneumonia can be achieved by choosing a method of immunosuppression that avoids parenteral administration (same comment for the choice of antibiotics prophylaxis) and by using parameters other than the overall mortality to assess the disease progression.

Studying articles published for the last 60 years has enabled us to establish a wide range of criteria and factors to be considered for implementing an animal model to address *Pneumocystis* pneumonia. This required making choices to best answer the question posed and included many elements, such as permissiveness to infection, homology, analogy, and fidelity with humans, as well as reproducibility, ease of handling, safety, and of course cost. Thus, if one wonders about the cycle of *Pneumocystis*, it seems more relevant to replicate the natural transmission of *Pneumocystis* in mammals by using co-housing of healthy animals with infected fellows, whether they be rodents, with which we have the most experience, or NHPs, whose *Pneumocystis* species is the closest phylogenetically to that of humans. In contrast, in pre-clinical therapeutic studies that require rigorous design to obtain a homogenous population, a model with an implementation of the infection by direct inoculation of *Pneumocystis* organisms allows necessary reproducibility and high control. For studies focusing on the understanding of the pathophysiology and particularly the host immune response, several types of models can be suggested. The first ones use refined, genetically modified mice with a very specific immunodeficiency to study its specific involvement in the host response. The other ones study the immune response more generally, using models displaying general immunodeficiency such as genetically modified SCID or RAG1-/- mice or animals immunosuppressed by the use of corticosteroids, the major iatrogenic risk factor of *Pneumocystis* pneumonia in humans [57,58,59].

**Figure 4:**
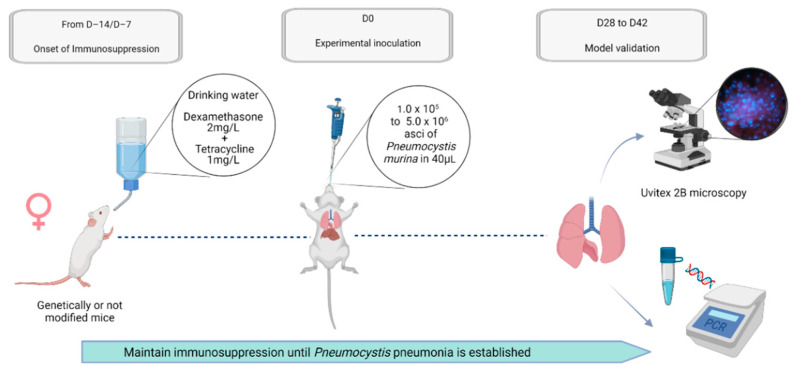
Example of a mouse model to explore the immune response during *Pneumocystis* pneumonia. The aforementioned suggestions are based on the analysis of the published literature faced with the authors’ personal experience. Considering all their benefits, including their small size, their costs, and the large availability of the toolbox dedicated to them, mice should be privileged. Depending on the purpose of the study, particularly for studies on the immune response, genetically manipulated strains can be used. Immunocompromised status is achieved by administration of steroids within drinking water during a 1–2-week-long period. In order to prevent undesirable opportunistic bacterial infection, antibiotics must be used. Thereafter, to control the source and the burden of *Pneumocystis*, the experimental infection will be completed by an intranasal challenge, ideally with an inoculum situated between 1.0 × 10^5^ and 5.0 × 10^6^ asci. Generally, in this model, the onset of clinical signs occurs within 4–6 weeks after the infectious challenge. Alternative endpoints to death may be assessed to validate the infection model and estimate the fungal load while refining the animal procedures. Microscopic observations of pulmonary secretions, lung sections, and lung grindings as well as molecular biology techniques appear reliable and largely validated.

This review, however, is subject to several limitations. The first one is the limited access to data and particularly to older studies. The second limitation is related to the exhaustiveness of our review. The study of all animal models of *Pneumocystis* pneumonia allowed us to highlight crucial parameters to be considered by the investigator, but it did not allow us to explore all specific cases in depth. Nevertheless, according to our experience, we can propose a relevant example of an animal model to study the immune response that uses genetically modified (or unmodified) and steroid immunosuppressed rodents, challenged by intranasal inoculation of *Pneumocystis murina* and validated by a microscopic and molecular biology technique (Figure 4). However, the scientific debate is not close to being finished.

## Figures and Tables

**Table 1:** Overall description of the main parameters considered in the selected published animal models of *Pneumocystis* pneumonia, according to the criteria reported in Figure 1.

	Mean (Unit ± Standard Deviation) or Number (%); 95% Confidence Interval				
	Mouse *N* = 560 (74.8%)	Rat *N* = 156 (20.8%)	Rabbit *N* = 10 (1.3%)	Non-Human Primate *N* = 10 (1.3%)	Other Animal *N* = 13 (1.7%)
Weight	21.0 (±4.5); (18.9–23.3 g)	189.4 g (±48.4); (181.8–197 g)	-	-	-
Sex					
- Males	36 (6.4%)	59 (37.8%)	-	2 (20%)	2 (15.4%)
- Both	28 (5%)	6 (3.8%)	1 (10%)	3 (30%)	-
- Undetermined	431 (77%)	30 (19.2%)	9 (90%)	5 (50%)	9 (69.2%)
Animal strains, *including:*					
- Outbred	14 (2.5%)	138 (88.5%)	10 (100%)	10 (100%)	13 (100%)
- Inbred	546 (95.5%)	18 (11.5%)	-		
Immunosuppressive regimens, *including ^φ^:*					
- Steroids	67 (12%)	150 (96.2%)	2 (20%)	1 (10%)	11 (84.6%)
- Immunotherapy	162 (28.9%)	2 (1.2%)	-	-	-
- Other immunosuppressive drug(s)	-	5 (3.2%)	-	1 (10%)	-
- Mutation deletion	330 (58.9%)	-	-	-	-
- Alternative method(s)	-	-	-	8 (80%)	-
Exposition, *including:*	N = 325 (58%)	N = 71 (45.5.%)	N = 5 (50%)	N = 4 (40%)	N = 2 (15.4%)
- Standard conditions	24 (4.3%)	32 (20.5%)	3 (30%)	2 (20%)	1 (7.7%)
- Microisolator-filtered cages	301 (53.8%)	39 (25%)	2 (20%)	2 (20%)	1 (7.7%)
Nutritionnal regimen	N = 323 (57.7%)	N = 87 (55.8%)	N = 3 (30%)	N = 7 (70%)	N = 2 (15.4%)
- Normal	297 (53%)	54 (34.6%)	3 (30%)	7 (70%)	2 (15.4%)
- Low-protein	26 (4.6%)	33 (21.2%)	-	-	-
Route of experimental infection inoculum size					
- Co-housing	104 (18.6%)	17 (10.9%)	-	5 (50%)	-
- Oropharyngeal instillation	28 (5%)	-	-	-	-
	2 × 10^5^		-	-	-
- Intranasal instillation	38 (6.8%)	2 (1.3%)	-	-	-
	6.0 × 10^6^ (±7.5 × 10^6^); (3.5–8.5 × 10^6^)	1.10^7^ (±1.4 × 10^7^); (0.0–3 × 10^7^)		-	-
- Transtracheal deposition	42 (7.5%)	29 (18.6%)	-	-	-
	4.8 × 10^6^ (±1.1 × 10^7^); (1.1–8.5 × 10^6^)	1.3 × 10^7^ (±3.1 × 10^6^); (0.1–2.5 × 10^7^)		-	-
- Intratracheal instillation	306 (54.6%)	17 (10.9%)	-	1 (3.3%)	1 (7.7%)
	6.3 × 10^6^ (±1.5 × 10^7^); (4.5–8.1 × 10^6^)	1.4.10^7^ (±2.7 × 10^7^); (0.1–2.6 × 10^7^)		5.10^6^	2.10^5^
- Without infection strategy	42 (7.5%)	91 (58.3%)	3 (100%)	4 (40%)	12 (92.3%)
Validation of the model and parameters to follow, *including* *^φ^:*					
- Microscopy	439 (78.7%)	146 (94.2%)	3 (100%)	6 (60%)	12 (92.3%)
- Serology	40 (7.2%)	7 (4.5%)	-	4 (40%)	1 (7.7%)
- Molecular biology	205 (36.7%)	19 (12.3%)	1 (33.3%)	8 (80%)	2 (15.4%)
- ß-D-glucan measurement	10 (1.8%)	1 (0.6%)	-	-	-

*^φ^* Associations are possible.

**Table 2:** Comparison of the four major animal models of *Pneumocystis* pneumonia (mouse, rat, rabbit, and non-human primate). These models are assessed here for their relative benefits and limitations. Relative scores are represented as being very good (green tick), partly suitable (yellow tick), and not suitable (red cross).

	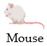	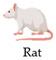	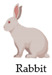	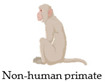
Relative cost	** ✓ **	** ✓ **	** ✓ **	**x**
Easy to breed	** ✓ **	** ✓ **	** ✓ **	**x**
Simplicity of maintenance and handling	** ✓ **	** ✓ **	** ✓ **	**x**
Study tools available	** ✓ **	** ✓ **	**x**	**x**
Tissue quantity available	** ✓ **	** ✓ **	** ✓ **	** ✓ **
Ethical restrictions	** ✓ **	** ✓ **	** ✓ **	**x**
Inbred strains and transgenic lines available	** ✓ **	** ✓ **	**x**	**x**
Immune response similarity to humans	**x**	**x**	**x**	** ✓ **
Anatomical, physiological, and genetic similarities to humans	** ✓ **	** ✓ **	** ✓ **	** ✓ **
Natural acquisition of *Pneumocystis* pneumonia	**x**	**x**	** ✓ **	**x**
Experimental acquisition of *Pneumocystis* pneumonia under virus-induced immunodepression	**x**	**x**	**x**	** ✓ **
Experimental acquisition *Pneumocystis* pneumonia under steroid-induced immunodepression	** ✓ **	** ✓ **	** ✓ **	** ✓ **

**Table 3:** Summary of the advantages and disadvantages of the principal strategies to render animals susceptible to *Pneumocystis* pneumonia and the main methods of experimental challenging to implement *Pneumocystis* pneumonia.

Model Type	Pros	Cons
**Strategies to render animal susceptible to *Pneumocystis* pneumonia**		
Steroids	Targeting T-cells and macrophages, largely involved in immune response against *Pneumocystis* spp. Major risk factor for the development of *Pneumocystis* pneumonia in humans Administrable in drinking water for some molecules (convenient, safe, compatible with refinement of experimental procedures)	Start 1–2 weeks prior to experimental inoculation or co-housing Need to be continuously pursued until the infection had been established Anti-inflammatory effects that can interfere with the immune response (confounding bias) Not representative of the viral induced-immunosuppression
Immunotherapy	Selective depletion of different cell types to evaluate their impact in the *Pneumocystis* pneumonia development Avoiding confounding bias seen with steroids	Administrable by injection (no refinement of experimental procedure) Start 1–2 weeks prior to experimental inoculation or co-housing Needs to be continuously pursued until the infection has been established Risk of hypersensitivity reaction or cytokine release-associated acute reactions Not exploring redundancy in the immune system or compensatory hyperactivity
Genetically modified animal	Selective depletion of different components of the immune response to evaluate their impact in *Pneumocystis* pneumonia development Recapitulating the human primary immune disorders Avoiding confounding bias seen with steroids Avoiding administration of drug to induce immunosuppression	Expensive Not exploring redundancy in the immune system or compensatory hyperactivity Restricted to specific models, especially mice Not representative of the viral induced-immunosuppression
Viral induced- immunosuppression	Evaluation of *Pneumocystis* pneumonia in a viral-induced immunosuppression context Avoiding administration of drug to induce immunosuppression Avoiding confounding bias seen with steroids	Restricted to comparisons in the context of viral induced-immunosuppression Possible only for non-human primates (ethical restrictions)
**Strategies to implement *Pneumocystis* pneumonia**		
Passive without co-housing (only based on immunosuppression induction)	No instillation procedure to be performed No index case animals to use	Not relevant to the transmission and cycle of *Pneumocystis* Lack of reproducibility Inoculum not known
Passive by co-housing	Close to natural transmission No intervention to be performed	Need to breed pre-infected mice in the laboratory Lack of reproducibility Inoculum not known
Active by instillation (oropharyngeal, intranasal, transtracheal, intratracheal)	Reproducibility Control of the timing of the infection Known inoculum	Inoculated microorganisms not pure because isolated from filtered lung shreds of infected animals, possible influence on immune response (need to control) Higher inoculum than in a natural transmission Need for anesthesia and intervention by trained personnel

## Data Availability

Not applicable.
